# Tissue Biocompatibility and Antimicrobial Properties of Sympathomimetic Nasal Solutions for Potential Use in Dental Pulpal Management

**DOI:** 10.1002/cre2.70259

**Published:** 2025-12-10

**Authors:** Robert S. Jones, Roman Garcia, Isha Mutreja, Dhiraj Kumar

**Affiliations:** ^1^ Division of Pediatric Dentistry, Department of Developmental & Surgical Sciences, School of Dentistry University of Minnesota Minneapolis Minnesota USA; ^2^ Impact Biomaterials Lab, Division of Restorative Science Minnesota Dental Research Center for Biomaterials and Biomechanics (MDRCBB), School of Dentistry University of Minnesota Minneapolis Minnesota USA; ^3^ Division of Basic Sciences, Department of Diagnostic and Biological Sciences, School of Dentistry University of Minnesota Minneapolis Minnesota USA

**Keywords:** anti‐bacterial agents [D27.505.954.122.085], biocompatible materials [D25.130], endodontics [E06.397], pediatric dentistry {H02.163.876.600}, sympathomimetics [D27.505.696.663.050.870]

## Abstract

**Objectives:**

Sympathomimetic nasal solutions (SNS), containing oxymetazoline or phenylephrine, may be used directly onto dental pulp tissue and initiate hemostasis via activation of alpha‐receptors that innervate arteriole smooth muscles. The aim of this study was to assess these SNS's biocompatibility with human dental pulp stem cells (hDPSCs) and ability to kill a deep caries‐associated pathogen, *Rothia dentocariosa*.

**Materials and Methods:**

A Kirby‐Bauer disk diffusion susceptibility test assessed SNS zones of inhibition (ZOI) toward *R. dentocariosa* growth on agar plates. Over‐the‐counter (OTC) SNS containing 0.025% and 0.05% oxymetazoline (NS‐OXY‐kids, NS‐OXY‐original) and 1% phenylephrine (NS‐PHE) were tested along with ingredients found in these nasal solutions: 0.025%, 0.05% oxymetazoline; 0.0125%, 0.025% benzalkonium chloride; and 0.2%, 1% phenylephrine (PHE). A second antimicrobial broth inhibition assay examined effects on *R. dentocariosa* planktonic growth. A 24‐h recovery biocompatibility assay with resazurin (alamarBlue) was performed on hDPSC that were exposed to pure respective compound solutions, NS‐OXY‐kids, NS‐PHE, and 1% PHE for 10 min.

**Results:**

NS‐OXY‐kids, NS‐OXY‐original, NS‐PHE, and 0.025% BKC were bactericidal toward *R. dentocariosa* with an average ZOI of (16.33 ± 2.22 [average ± SD] mm). NS‐PHE had a small but statistically significant difference (17.83 ± 3.189 mm) between NS‐OXY‐kids (14.67 ± 1.033 mm); 0.0125% BKC had a smaller (*p* < 0.05) ZOI of 10.5 ± 0.837 mm. Solutions containing solely OXY (0.025%, 0.05%) and PHE (0.2%, 1%) demonstrated no bactericidal activity but had a slight bacteriostatic inhibition in the broth inhibition assay. The commercial OTC formulations of NS‐PHE and NS‐OXY‐kids needed to be diluted 1:10 to have near 50% metabolic activity of hDPSCs normalized to untreated control cells.

**Conclusions:**

NS‐PHE and NS‐OXY (both original and kids) had antimicrobial activity toward *R. dentocariosa* and comparable biocompatibility with hDPSCs. SNS may have both hemostatic and antimicrobial properties if used as pulpal medicaments.

## 1 Introduction

The practice of vital pulpal therapy is gaining traction as a viable alternative to full pulpal extirpation in more advanced cases of pulpitis (Gadallah et al. 2024; Li et al. 2019). For several years, the effectiveness of indirect pulp capping questioned the overuse of pulpotomies in cases of asymptomatic deep caries in primary and permanent teeth (Smaïl‐Faugeron et al. 2016). However, evaluating the evidence on the treatment of irreversible pulpitis has led to a reexamination into both the diagnosis of the term “irreversible” and the treatment of teeth with symptoms of pain, both short‐lived and lasting (Ather et al. 2022). Long‐term success of vital pulp therapy in the treatment of symptomatic teeth is attributed to pulpal inflammation being mainly confined to the coronal pulp space and calcium silicate pulp capping materials modulating the immune response favorably (Chang et al. 2014; Ricucci et al. 2014). Research is also reassessing the long‐standing practice of evaluating pulpal health status by measuring the duration of time to achieve pressure hemostasis. While it seems reasonable to accept hemostasis as a definitive clinical assessment tool, a recent study didn't show that inflammatory mediators are correlated to the duration of time to achieve hemostasis (Mutluay et al. 2018). A plausible hypothesis is that excess bleeding may be associated with pro‐inflammatory pathways that are creating edema and recruiting immune cells. In other studies, duration to achieve pressure hemostasis was poorly correlated to the outcome of the pulpotomy procedure (Lai et al. 2024; Waterhouse et al. 2002). Assessing hemostasis has equivocal diagnostic value despite being widely used by clinicians (Almutairi 2024).

While the duration to achieve pressure hemostasis may not directly assess the state of pulpal inflammation, achieving hemostasis is needed prior to the application and setting of a pulp capping material (Hilton 2009). There are additional benefits of achieving effective hemostasis that reduces the blood clot volume. Large blood clots may reduce and slow pulpal healing and harbor bacteria and debris (Hørsted et al. 1981). The result may reduce the intimate contact between pulpal tissue and a biocompatible pulp capping material. With the inclusion of pulps that have prolonged bleeding times into vital pulpal therapy practice, there is a need for effective hemostatic agents that have dual antimicrobial activity (Dronamraju et al. 2023).

Current formulations of nasal decongestant sprays contain various sympathomimetic compounds that can activate alpha‐adrenergic receptors and initiate topical hemostasis (Haenisch et al. 2010). Although the primary use of decongestant sprays has been to relieve nasal congestion and reduce tissue edema, sympathomimetic‐based nasal sprays (SNS) with oxymetazoline (NS‐OXY) are routinely used during otorhinolaryngology surgical procedures and by anesthesia teams to reduce nasal bleeding during intubation (R. S. Cartabuke et al. 2019; Higgins et al. 2011; Masoudifar et al. 2023; Rodriguez Valiente et al. 2013). To a lesser extent, topical phenylephrine, also a sympathomimetic compound, has been used to manage bleeding (Riegle et al. 1992). SNS of phenylephrine (NS‐PHE) and NS‐OXY are available over‐the‐counter (OTC) for children and adults. As a SNS for children 12 years of age and above, 1% NS‐PHE is a highly effective surgical hemostatic agent, but due to its potency can produce systemic arterial vasoconstriction and reflex bradycardia, especially in undefined soaking of nasal pledgets (Higgins et al. 2011). On the other end of the spectrum, a young children's formulation (ages 2 to under 6) of NS‐OXY (0.025% OXY) carries much lower systemic risk (Higgins et al. 2011). Despite their use in medicine, topical SNS are not widely used off‐label to manage pulpal tissue bleeding. The existence of such FDA approved clinical available drugs or formulations leaves scope for the potential repurposing and application of these in dentistry and or elsewhere based on the properties of active and or inactive components.

The dental pulp is innervated primarily by the autonomic nervous system and vasoconstriction is mediated by the activation of alpha‐receptors (Olgart 1996). Sympathomimetic compounds, such as PHE and OXY, can activate alpha‐receptors and initiate hemostasis within the dental pulp (Haenisch et al. 2010). With contemporary evidence indicating the duration of bleeding to achieve pressure hemostasis is not a reliable diagnostic indicator of pulpal health, bleeding control aided by sympathomimetic‐based agents that reduce blood clot volume may have clinical utility (Jones 2021).

In this work, SNS underwent biocompatibility testing with human dental pulp stem cells (hDPSCs) and these solutions were diluted to examine the dose‐response. Since antimicrobial activity may also aid in managing carious pulp exposures and pro‐inflammatory pulpal response (Tüzüner et al. 2012), the bactericidal activity of SNS against *Rothia dentocariosa*, a pathogen associated with deep dentine caries, was evaluated.

## 2 Materials and Methods

### 2.1 Materials

*Rothia dentocariosa* (ATCC 17931) was purchased from ATCCC and Cytiva Whatman, 05‐711, 6 mm Disc inhibition assay discs were purchased from Fisher Scientific. Afrin Kids (NS‐OXY‐kids), Afrin original (NS‐OXY‐original), and 1% phenylephrine (NS‐PHE) were purchased from Amazon USA. hDPSCs (PT‐5025) were purchased from Lonza USA and were grown as per the supplier instructions. Oxymetazoline (OXY) powder was purchased from Cayman Chemical, USA, pure phenylephrine hydrochloride powder (AST‐D7003‐25G) was ordered from Neta Scientific and pure benzalkonium chloride (BKC) was purchased from Millipore Sigma USA. The pure compounds and other compounds were stored as per std conditions by suppliers and the different dilutions were prepared in commercially available double distilled water to avoid any contamination.

### 2.2 Methods

#### 2.2.1 Antimicrobial Susceptibility Testing

*Rothia dentocariosa* (ATCC 17931) cultures were grown aerobically overnight at 37°C in brain heart infusion (BHI) media. A Kirby‐Bauer disk diffusion susceptibility test was used to assess the sensitivity or resistance of select treatment compounds toward *R. dentocariosa*. BHI‐agar plates were prepared and 300 µL of *R. dentocariosa* bacterial inoculum (OD, 0.5 at 600 nm) was dispersed with an L‐shaped disposable spreader. The inoculated BHI‐agar plates were left to dry for 10–15 min at room temperature in a sterile environment. All solutions including the BHI growth media (sterilized by autoclave) were prepared in distilled water to control/minimize inorganic phosphate contamination. Commercial formulations of the adult and children's dosing NS‐OXY (Afrin original and kids, Bayer Healthcare, pH = 5.86) and adult 1% NS‐PHE (Amazon Basic Care, pH = 6.29) were evaluated (Table 1). Custom 0.025% and 0.05% oxymetazoline (OXY) solutions were prepared from pure compound OXY powder (Cayman Chemical, USA, stored as per supplier instructions) with a pH of 4.6. A custom solution of 1% PHE was prepared from pure phenylephrine hydrochloride powder (AST‐D7003‐25G, Neta Scientific, stored as per supplier instructions) with a pH of 5.91. To assess the antimicrobial effects of the preservatives in both NS‐OXY and NS‐PHE, custom solutions of 0.025% and 0.0125% benzalkonium chloride (BKC, Millipore Sigma USA, stored as per supplier instructions), also known as alkylbenzyldimethylammonium chloride, were prepared with distilled water (pH = 4.70). The BKC mixture used different chain length alkylbenzyldimethylammonium chlorides: approximately 70% benzyldime‐thyldodecylammonium chloride CH3(CH2)11N(Cl)(CH3)2CH2C6H5, approximately 30% benzyldimethyltetradecylammonium chloride CH3(CH2)13N(Cl)(CH3)2CH2C6H5. All the solutions were stored at 4°C until used at room temperature; 20 µL solutions of each treatment group were added to specialized porous discs (*n* = 6, Cytiva Whatman, 05‐711, 6 mm) and allowed to dry for 10 min. The discs were then gently pressed against BHI‐agar plates with surface colonies of *R. dentocariosa*. Plates were incubated (37°C) in an aerobic chamber for 20 h.

**Table 1:** Sympathomimetic‐based nasal solutions.

Product	Active ingredient	FDA approved ages (years)	Key inactive ingredients^a^	Other inactive ingredients^a^
NS‐OXY‐original (Afrin original)	0.05% oxymetazoline HCL	≥ 12	Benzylalkonium chloride (BKC)	Edetate disodium, polyethylene glycol, povidone, propylene glycol, purified water, sodium phosphate dibasic, sodium phosphate monobasic
		6–12		
		(supervised)		
NS‐OXY‐kids (Afrin children's)	0.025% oxymetazoline HCL	2‐under 6	Benzylalkonium chloride (BKC)	Dibasic sodium phosphate, edetate disodium, glycerin^b^, monobasic sodium phosphate, polyethylene glycol, povidone, propylene glycol, purified water
NS‐PHE (Amazon basic care)	1% Phenylephrine HCL	≥ 12^c^	Benzylalkonium chloride (BKC)	Benzyl alcohol, boric acid, purified water, sodium borate

^a^ Concentration of inactive ingredients is proprietary for each product.

^b^ Afrin children's is labeled as a moisturizing formulation with glycerin.

^c^ Under 12 ask your doctor.

In a separate assay that assessed planktonic growth inhibition, treatment group solutions (*n* = 6) were mixed with BHI media to achieve concentrations used in the disc susceptibility assay. Commercial formulations were unable to be pre‐concentrated so these compounds were diluted (1:5). *R. dentocariosa* were grown aerobically at 37°C in BHI broth and after measuring OD of the culture. A pre‐calculated volume of bacterial suspension was added to culture tubes at an initial OD of approximately 0.15 at 600 nm. A serial experiment of OD assessment was performed over 8 h to assess growth inhibition effects of treatment solutions.

### 2.3 Human Cell Viability Testing

hDPSCs, which were initially isolated from adult third molars, were purchased from Lonza Biosciences (hDPSCs, PT‐5025) and stored at −80°C prior to culturing the cell line. A supplied kit medium was used to initially grow the cells, followed by culture in minimal essential media (α‐MEM, Gibco, Thermo Fisher) supplemented with 10% fetal bovine serum (FBS) and 1% penicillin‐streptomycin (Pen‐Strep). Treatment groups for the biocompatibility study include 0.25% oxymetazoline (NS‐OXY‐kids, Children's strength Afrin, Bayer Healthcare), 1% phenylephrine hydrochloride (NS‐PHE, Amazon Basic Care), and custom formulations of 0.1%, 0.5%, and 1% phenylephrine hydrochloride (PHE powder, Neta Scientific). NS‐OXY‐kids and NS‐PHE were diluted to a specific concentration using the cell culture media, while the 1% PHE solution was first prepared in DI water followed by dilution in cell culture media. hDPSCs (10,000 cells per well, *n* = 6) were exposed to the treatment groups (two sets) at standard and diluted concentrations for 10 min, which simulated a long clinical exposure time in the dental clinic. After 10 min, hDPSCs in each well (both sets) were rinsed with 1× sterile PBS solution. Set 1 plates were used for the metabolic activity assessment at 10 min by following the standard alamarBlue assay protocol as per manufacturer's instructions (Kumar et al. 2025). While the set 2 plates were prepared for metabolic assessment after 24 h. The 24 h exposure assessment measured cellular recovery from the 10 min treatment exposures. For 24 h, hDPSCs were incubated in α‐MEM media with 10% FBS and 1% Pen‐Strep at 37°C in a humidified air incubator with 5% CO_2_ post 10‐min exposure and washing. The alamarBlue (Thermo Fisher) cell viability assay testing was repeated after 24 h on treated hDPSCs. Fluorescence measurements (540‐nm excitation/590‐nm emission, Synergy HT, Biotek, VT, USA) for both periods were measured.

### 2.4 Statistical Analysis

Statistical analysis was performed using ANOVA and post hoc testing for pairwise differences using Prism (GraphPad, Boston, MA). The error bars in the respective graph are represented as 95% confidence interval.

## 3 Results

**Figure 1 (A):**
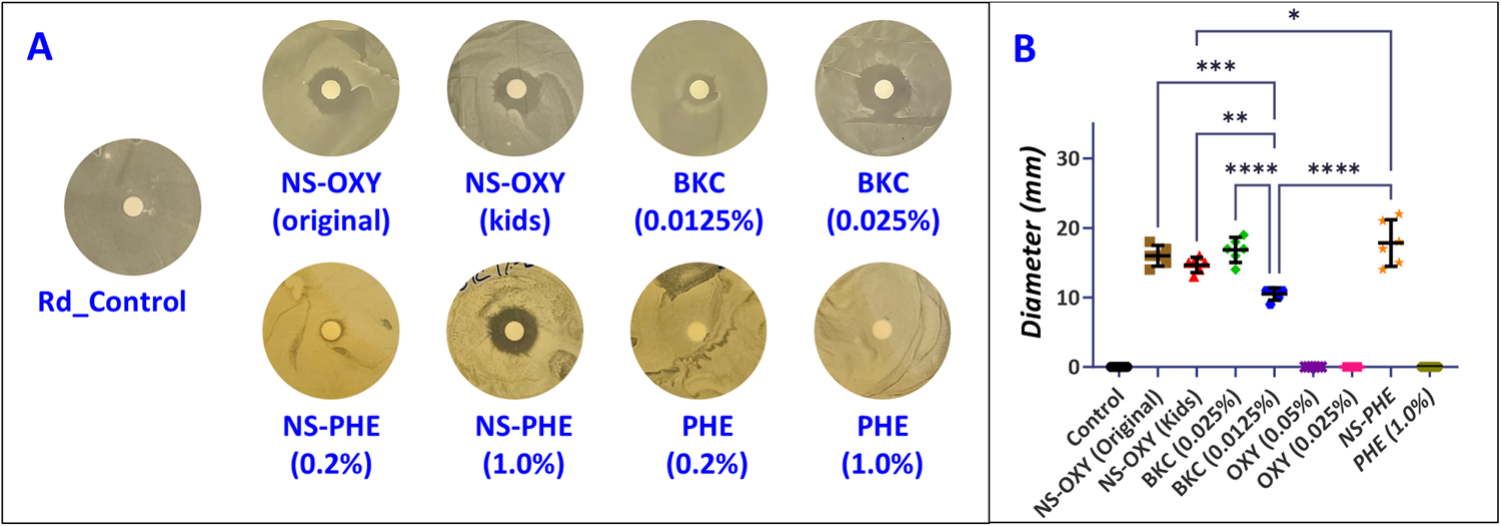
Disk diffusion susceptibility testing of test solutions against *Rothia dentocariosa* after 20 h of incubation. OXY, PHE, and BKC were also tested. (B) The diameter of the zone of inhibition around each disc was measured (*n* = 6). Larger diameters of inhibition indicated that the test group diffused farther into the colony and created a no growth region. There was no statistical difference found between NS‐OXY‐adults, NS‐OXY‐child, NS‐PHE, and 0.025% BKC. Mean and 95% CI shown. There were differences between groups and 0.0125% BKC. (* = *p* ≤ 0.05, ** = *p* ≤ 0.01, *** = *p* ≤ 0.001, and **** = *p* ≤ 0.0001).

**Figure 2:**
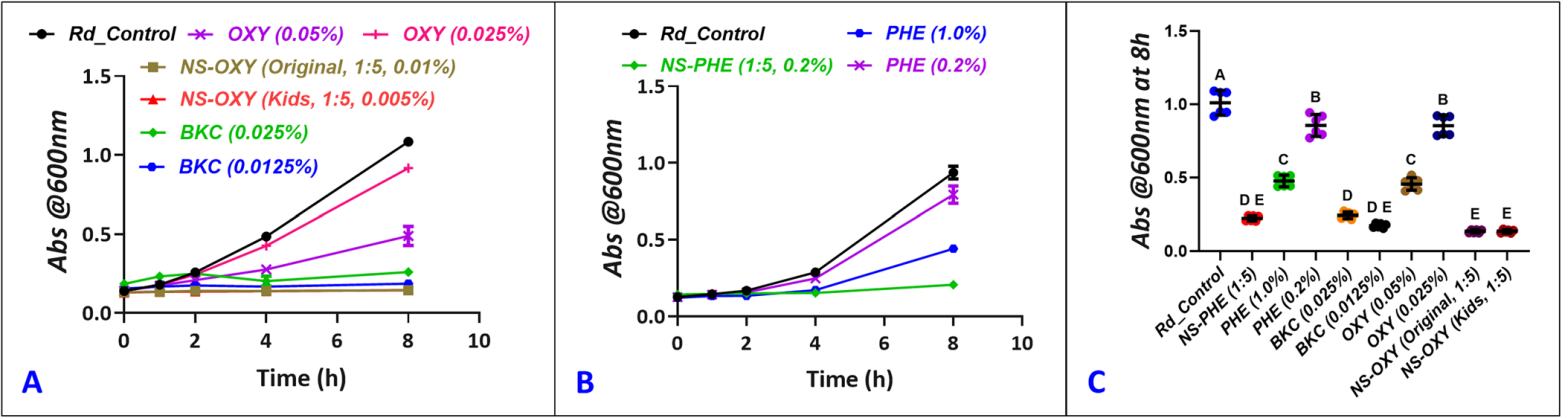
Stock NS‐OXY original—0.05% Oxy (adults), NS‐OXY Kids—0.025% Oxy, and NS‐PHE—1% phenylephrine hydrochloride. Planktonic growth of *Rothia dentocariosa* in BHI media as measured with optical density (OD) absorbance at 600 nm. Bacterial OD values were serially measure up to 8 h from inoculation. (A) Growth of bacteria treated with NS‐OXY (original, adults, 1:5, 0.01% Oxy), NS‐OXY (kids, 1:5, 0.005% Oxy), OXY (0.025%, 0.05%), BKC (0.0125%, 0.025%), and untreated control. (B) Growth of bacteria treated with 0.01% NS‐PHE, PHE (0.2%, 1.0%), and untreated controls (*n* = 6). C) *Rothia dentocariosa* planktonic growth comparison between all treatment groups after 8 h. All treatment groups showed a reduction in optical density versus the untreated control group (ANOVA, post hoc Dunnett's). Mean and 95% CI shown. Pairwise analysis was performed post hoc (ANOVA, Tukey–Kramer) with different letters designating *p* < 0.05.

In the disc diffusion susceptibility test, NS‐OXY (kids, original), NS‐PHE, and 0.025% BKC (0.025%) had substantial antimicrobial activity against *R. dentocariosa* (Figure 1A). Bactericidal activity was visualized by a clear zone around the treatment‐impregnated discs, which indicated a zone that lacked bacterial growth on the agar plate. No difference in the zone of inhibition (unit: mm ± SD) was measured between NS‐PHE (17.83 ± 3.19), NS‐OXY‐adult (16.0 ± 1.41), and 0.025% BKC (16.83 ± 1.72) (Figure 1B). NS‐PHE (17.83 ± 3.19) had a slightly larger zone of inhibition versus NS‐OXY‐kids (14.67 ± 1.03) (*p* < 0.05). However, each of these treatments had statistically higher antimicrobial activity than the diluted BKC concentration of 0.0125% BKC (10.51 ± 0.83). Distilled water, 1% PHE, and both concentrations of OXY (0.025% and 0.05%) had no antimicrobial activity toward *R. dentocariosa* on BHI agar (Figure 1; Figure S1). Broth inhibition assay demonstrated that OXY had a slight inhibitory effect on *R. dentocariosa* growth with 0.05% demonstrating greater inhibition than 0.025% (Figure 2A). Phenylephrine also attenuated *R. dentocariosa* growth with 0.2% exhibiting a negligible but statistically significant reduction and 1% having modest reduction in growth (Figure 2B). Pure concentrations of OXY and PHE did not reduce growth as substantially as NS‐OXY (kids, original), NS‐PHE, and BKC (0.0125% and 0.025%) as shown in Figure 2A–C. Both concentrations of BKC demonstrated near total growth inhibitory effects. NS‐OXY and NS‐PHE were commercial formulations and could not be pre‐concentrated, so the clinical formulations were diluted (1:5) as required. In summary for the planktonic growth inhibition assay, all treatment groups (Figure 2C) showed a reduction in optical density versus the untreated control group (ANOVA, post hoc Dunnett's).

**Figure 3:**
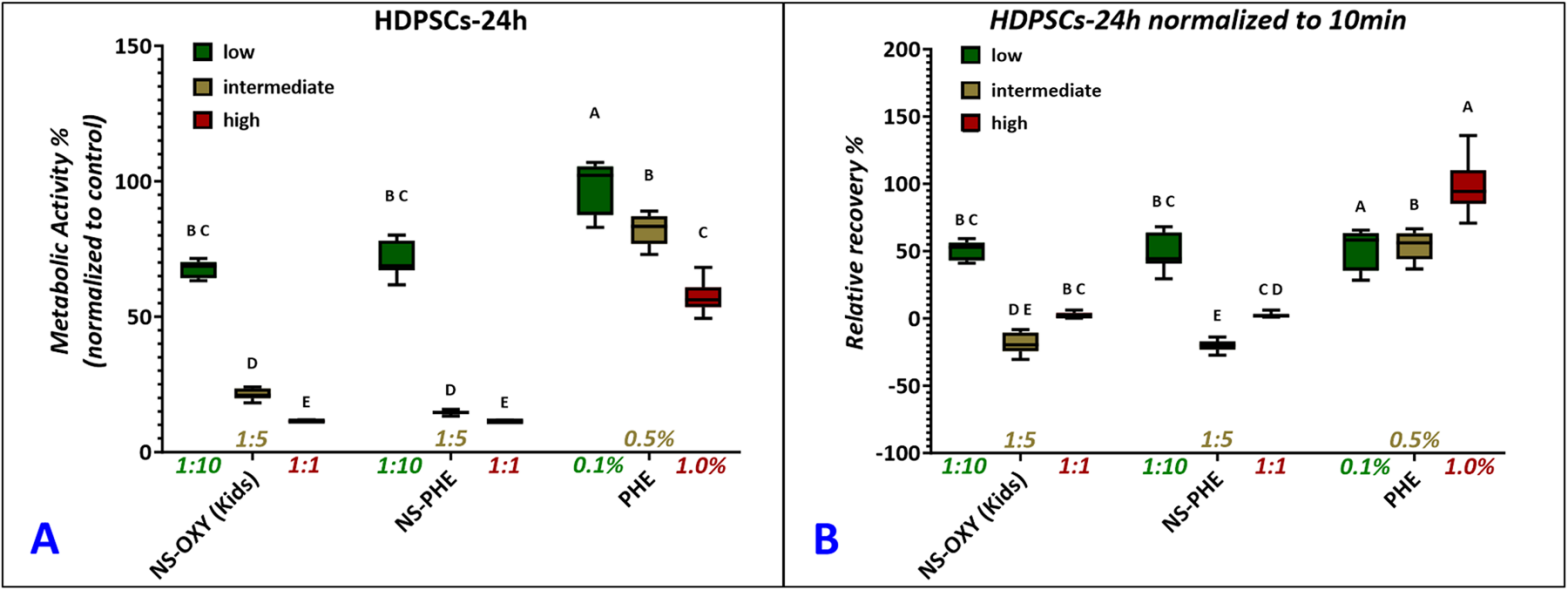
Metabolic activity (assessed using alamarBlue assay, *n* = 8) of human dental pulp stem cells (hDPSCs) when exposed to each reagent and dilution for 10‐min shock treatment and allowed to recover in cell culture media for 24 h (A) and metabolic activity at 24 h recovery normalized to 10 min exposure activity showing the recovery of hDPSCs post treatment (B). Pairwise analysis was performed post hoc (ANOVA, Tukey‐Kramer) with different letters designating *p* < 0.05. Box (mean, 95% CI) and whisker (data range) plots are shown.

For the biocompatibility assessment, unaffected hDPSCs control samples, which were exposed to distilled water, showed high cellular metabolic activity which is also a sign of cellular viability by the substantial reduction of resazurin‐based molecules in the alamarBlue assay. In general, metabolic activity below 60% is considered to have low to no biocompatibility. In our study, the treatment groups of NS‐OXY‐kids, NS‐PHE, and 0.1%, 0.5%, and 1% PHE were used for a treatment of 10 min followed by recovery of hDPSCs in the cell culture media for 24 h. The 10 min duration translated to exposure of cells to the treatment chemicals, drugs, or formulations during the pulp management procedure. The metabolic activity at 24 h normalized to the untreated control group and the relative recovery of hDPSCs (metabolic activity normalized to the 10 min activity of the same group) are shown in Figure 3A,B, respectively. The hDPSCs exposed to PHE at 0.1% and 0.5% reduced resazurin to resorufin by 98.48% and 82.36%, respectively; 1% PHE reduced hDPSCs to 57.23%, suggesting low biocompatibility at 1% phenylephrine concentration. However, the relative recovery percentage data suggested, in the case of pure phenylephrine, that HDPSCs recovered by more than 55% (Figure 3B). In addition, only the lowest concentration (1:10) of NS‐OXY‐kids and NS‐PHE showed biocompatibility with an average metabolic activity of 67.54% and 71.22% at 24 h. The relative recovery % data suggested less than 50% recovery at the lowest treatment concentration for both. The higher concentrations (1:5, 1:1) of both NS‐OXY‐kids and NS‐PHE reported no recovery in metabolic activity of hDPSCs post 10‐min treatment after 24 h.

## 4 Discussion

The present study establishes initial biocompatibility with hDPSCs and antimicrobial activity toward *R. dentocariosa* profiles for NS‐PHE and NS‐OXY‐kids. These sympathomimetic nasal solutions (SNS) have potential off‐label use as direct topical hemostatic agents on pulp tissue. The dental pulp is composed of both alpha‐1 and alpha‐2 receptors, and both phenylephrine and oxymetazoline activate these receptors with different specificity (Deol et al. 2023; Haenisch et al. 2010). While both sprays are expected to produce hemostasis via alpha‐receptor activation (Gupta et al. 2012; Higgins et al. 2011), future work will investigate pulpal hemostasis efficacy between the different compounds. Based on the results of the present study, no differences between NS‐PHE and the NS‐OXY‐kids were found in tissue compatibility with hDPSCs. Both compounds exerted substantial bactericidal activity against *R. dentocariosa* with NS‐PHE exhibiting a slightly higher activity. NS‐OXY‐kids formulation had near identical tissue biocompatibility, measured by resazurin‐based molecule reduction, as found previously with NS‐OXY original formulation (Kumar et al. 2025).

The similarities in the tissue biocompatibility and antimicrobial profiles of NS‐OXY and NS‐PHE are likely the result of the ddition of proprietary concentrations of BKC in both nasal solution bottles. BKC is a preservative within many consumer products that prevents bacterial growth (Chen et al. 2018). In the case of nasal solutions, bacteria from the nasal cavity can contaminate the inner solution of the bottle, especially when used as a repeated spray for nasal decongestion. For this reason, BKC is added to nasal solutions to prevent bacterial overgrowth (Graf et al. 1999). This work estimated the BKC chain length distribution based on ideal water solubility. The concentration was also estimated to be 0.025% in the bottle (Jones 2021). In commercial OTC sprays such as NS‐OXY and NS‐PHE, the concentration of BKC is proprietary within commercial OTC sprays. NS‐OXY and NS‐PHE had similar antimicrobial activity to the 0.025% BKC that was used in this study. The direct consequence of having antimicrobial activity toward *R. dentocariosa* and other bacterial pathogens is that NS‐OXY and NS‐PHE can be used to cleanse and disinfect the pulpal canal space while also providing potential hemostasis. BKC has been shown in other studies to be a potent antimicrobial that has broad antimicrobial activity and substantivity by binding to demineralized tissue (Tezvergil‐Mutluay et al. 2011; Watrobska–Swietlikowska 2019). In the case of NS‐PHE and NS‐OXY, which both contain proprietary levels of BKC, these nasal solutions also contain additional inactive ingredients that act as preservatives. NS‐OXY contains BKC with edetate disodium (EDTA). EDTA improves the bacterial membrane disruption activity of BKC (Richards and Cavill 1976). In dental applications, EDTA is a chelating agent that can remove the smear layer and residual organic material (de Andrade Marafiga et al. 2022). NS‐PHE contains BKC with the additional preservatives of benzyl alcohol, boric acid, and sodium borate. These additional compounds, which are not found in NS‐OXY‐kids, may be the reason for the small difference in antimicrobial activity.

However, no difference in biocompatibility was measured between NS‐PHE and NS‐OXY‐original, which also does not have benzyl alcohol, boric acid, and sodium borate. While the exact BKC concentration in NS‐OXY and NS‐PHE is proprietary, the near equal biocompatibility suggests that either their BKC concentrations are similar or they are different and the addition of the other preservatives equalizes their overall biocompatibility. Previous work demonstrated that BKC even at lower concentrations of 0.0125% attenuated hDPSCs metabolic enzymatic activity in reducing resazurin based dyes (Kumar et al. 2025).

In the present study, PHE alone showed high biocompatibility. This is similar to previous work demonstrating OXY alone had high biocompatibility (Kumar et al. 2025). The commercial formulation of NS‐PHE and NS‐OXY‐kids had to be diluted 1:10 to have near 50% metabolic activity normalized to untreated control cells. This effect is similar to those seen in NS‐OXY‐original exposure to hDPSCs (Kumar et al. 2025). Given that a pulp medicament will be applied topically and dissipate into the pulpal space, these solutions are diluted through a diffusion‐mediated process. Furthermore, it may be recommended, based on the results of this present study, that excess NS‐OXY and NS‐PHE should be removed by cotton pellets at the pulpal access site to allow effective hemostasis without excess residual solution that affects deeper pulp tissue.

The 1:10 dilution of NS‐OXY‐kids and NS‐PHE that reduces the metabolic activity of hDPCS by 50% can be compared to literature values on the biocompatibility of sodium hypochlorite (Maru et al. 2022). There is a 55% reduction in the metabolism of dental pulpal stem cells with 0.005 mg/mL, which is a 1:1000 dilution of 5.25%, of sodium hypochlorite (Maru et al. 2022). In a recent previous study investigating NS‐OXY original formulation, the antimicrobial activity of full strength NS‐OXY against *R. dentocariosa* has been shown to be comparable to a 1:10 dilution of 5.25% sodium hypochlorite (Kumar et al. 2025). A near 1:10 dilution of 5.25% sodium hypochlorite will cause over 70% decrease in pulpal cell viability, suggesting high cytotoxicity in hDPSCs. Together these results suggest that NS‐OXY and NS‐PHE may have a better biocompatibility profile than sodium hypochlorite, but more work is needed to assess antimicrobial activity across different oral bacterial species and on dentin surfaces. EDTA is highly biocompatible with dental pulp cells and has the potential to be bio‐inductive toward mineralization via transforming growth factor‐β1 (TGF‐1β) release from the dentin matrix (Liu et al. 2021). The addition of BKC at lower concentrations (0.008%) to 17% EDTA, did not change TGF‐1β release and hDPSCs attachment levels when placed on dentin disks, which were later rinsed with water (Kucukkaya Eren et al. 2021). BKC, which is found in both NS‐OXY and NS‐PHE, has been shown to substantially reduce hDPSCs viability at concentrations of 0.0125% (Kumar et al. 2025). While the concentration of boric acid is unknown in the proprietary concentrations of NS‐PHE, hDPSCs maintain viability even at high boric acid concentrations (Kotan and Uysal 2024).

Research on the application of SNS in pulp therapy is relatively new compared to several decades of research on sodium hypochlorite. Future studies could examine if OXY and PHE can be mixed with other antimicrobial preservative compounds to improve biocompatibility while maintaining high antimicrobial activity. Additionally, future studies should directly compare sodium hypochlorite biocompatibility with NS‐OXY and NS‐PHE. Since the present study investigates the activity toward planktonic bacteria, future studies are needed to examine antibiofilm effects, which may more accurately predict clinical efficacy of SNS. The study used hDPSC for initial testing of biocompatibility since these third molar derived cells are commercially available, thus ensuring future reproducibility, and according to the manufacturer's information, the cell line has exhibited comparable phenotype expression as bone‐marrow derived mesenchymal cells. The present study has limitations in predicting the clinical response to SNS. 2D cell systems may underestimate biocompatibility since 3D systems have been shown to have reduced sensitivity to drug toxicity (Edmondson et al. 2014). 3D systems can limit the diffusion of drugs and possess protective connective tissue. In the case of BKC, the present study may overestimate the toxicity of BKC, since systemic clinical based reviews support that BKC is a safe preservative (Kawabata et al. 2016; Marple et al. 2004). Future work is needed to examine the effects of NS‐OXY on hDPSC differentiation, mature dental pulp cells, and an immune response.

While there is limited data to assess the systemic effects of these medications in dental applications, SNS use in otorhinolaryngology surgical procedures is suggestive of systemic safety in adults and young children (R. S. Cartabuke et al. 2019; Higgins et al. 2011; Masoudifar et al. 2023; Riegle et al. 1992; Rodriguez Valiente et al. 2013). NS‐OXY is more widely used in medicine than NS‐PHE for surgical procedures. In sinus surgery, NS‐PHE has higher systemic morbidity risk compared to NS‐OXY (Higgins et al. 2011). It is estimated that as little as 1 drop approximately 0.05 mL of NS‐OXY original (0.05% OXY) is required for effective hemostasis in a primary tooth pulpotomy (Jones 2021). This amount is substantially less than the volume (1–2 mL) associated with adverse hemodynamic effects of these nasal sprays in home and surgical settings in young children (Ramesh et al. 2013; Thrush 1995). Providers should be aware that undefined soaking of nasal pledgets with NS‐OXY original placed into young children during surgical has resulted in severe hypertension with reflex bradycardia (Latham and Jardine 2013). The true systemic risk with NS‐PHE and NS‐OXY usage in a dental pulpal application is unknown. The dilutions used in this study ranged from 1:2, 1:5, 1:10 to examine the dose response of the inactive ingredients on antimicrobial activity and cell activity. In terms of clinical application, this is comparable to applying 0.05–0.5 mL onto the pulp, which can be delivered via a dropper or syringe.

The present study suggests that NS‐PHE has slightly higher antimicrobial activity and equivalent biocompatibility than NS‐OXY‐kids. This suggests that as dentistry investigates the potential use of OTC SNS and considers potential safety, NS‐OXY‐kids and NS‐OXY‐original may be a first line candidate to investigate hemostatic effects in a clinical application. This also allows the potential repurposing of FDA approved drugs/formulation for additional clinical applications after systematic in vitro, in vivo and/or clinical trials to reduce the time from bench to bedside use.

The results of this study are bias to the specific nasal spray brand formulations tested. Since the inactive ingredient formulations are proprietary, the results of this present study may not represent the activity and effects of comparable SNS solutions with comparable active ingredient concentrations. The OXY concentration is lower in NS‐OXY‐kids (0.025%) than NS‐OXY‐original (0.05%). NS‐OXY‐kids is approved for use in children 2 to under 6 years of age, and the original version (0.05%), recommended for adults and children 6 years old and above. No difference was found in the antimicrobial and biocompatibility profile between the two NS‐OXY formulations. From these results, the inactive ingredient formulation may be the same. Nasal surgical interventions have used the NS‐OXY‐original in children under 6 years of age (R. Cartabuke et al. 2021; R. S. Cartabuke et al. 2019). Nasal surgical procedures have used substantially higher volumes than estimated for a dental pulpal application. It is unknown at this time what the optimal volume required to gain hemostasis with the two formulations of NS‐OXY. If the NS‐OXY‐kids formulation of NS‐OXY is found to require a slightly larger volume than NS‐OXY‐original to achieve the same level of hemostasis, the dental pulp tissue will be exposed to a greater amount of inactive preservatives such as BKC that, based on this present study, may cause localized cytotoxicity in the tissue.

In conclusion, NS‐PHE and NS‐OXY (both original and kids) had antimicrobial activity toward *R. dentocariosa* and comparable biocompatibility with hDPSCs. Future in vivo testing of these SNS can assess potential hemostasis when used as a pulp medicament, but assessing antimicrobial activity during patient care is limited. The importance of this current *in vitro* work is that NS‐PHE and NS‐OXY (both original and kids) have a potential secondary mechanism of antimicrobial activity toward a deep caries pathogen. Additional studies are needed to investigate the volume application of NS‐OXY children's and original formulation to manage the hemostasis of dental pulp tissues and whether rinsing is necessary to improve pulpal biocompatibility.

## Author Contributions

Conceptualization: Robert S. Jones and Dhiraj Kumar. Methodology: Robert S. Jones, Dhiraj Kumar, and Isha Mutreja. Formal analysis: Robert S. Jones, Dhiraj Kumar, Isha Mutreja, and Roman Garcia. Investigation: Roman Garcia, Dhiraj Kumar, and Isha Mutreja. Data curation: Roman Garcia, Dhiraj Kumar, and Isha Mutreja. Writing – original draft preparation: Robert S. Jones and Dhiraj Kumar. Writing – review and editing: Robert S. Jones, Dhiraj Kumar, and Roman Garcia. Visualization: Robert S. Jones and Dhiraj Kumar. Supervision: Robert S. Jones and Dhiraj Kumar. Project administration: Robert S. Jones and Dhiraj Kumar. All authors have read and agreed to the published version of the manuscript. All authors made substantial effort on this work.

## Conflicts of Interest

The authors declare no conflicts of interest.

## Data Availability

The data that support the findings will be available in the searchable Data Repository for University of Minnesota (DRUM), https://doi.org/10.13020/GZ81-4K47.
